# CHANGING PATTERN OF NON ALBICANS CANDIDEMIA: OCCURENCE AND SUSCEPTIBILITY PROFILE IN AN INDONESIAN SECONDARY TEACHING HOSPITAL

**DOI:** 10.21010/Ajidv18n2S.2

**Published:** 2024-07-04

**Authors:** WIWING Veronica, SURYADINATA Neneng, LUMBUUN Nicolaski

**Affiliations:** 1 Department of Microbiology, Faculty of Medicine, University of Pelita Harapan, Tangerang, Indonesia, Jendral Sudirman Boulevard, Lippo Karawaci, Tangerang, Banten, Indonesia 15811; 2 Faculty of Medicine, University of Pelita Harapan, Tangerang, Indonesia, Jendral Sudirman Boulevard, Lippo Karawaci, Tangerang, Banten, Indonesia 15811; 3 Department of Pharmacology, Faculty of Medicine, University of Pelita Harapan, Tangerang, Indonesia, Jendral Sudirman Boulevard, Lippo Karawaci, Tangerang, Banten, Indonesia 15811

**Keywords:** *Candida parapsilosis*, blood stream candidiasis, susceptibility, antifungal

## Abstract

**Background:**

In recent years, bloodstream infections due to non-*albicans* *Candida species* have been reported significantly among hospitalized patients, mainly among immunocompromised patients with high morbidity and mortality rates. A better understanding and awareness regarding the shift of *Candida albicans* flora to non-*albicans Candida species* is essentially important to improve treatment outcomes. In this study, we evaluated the distribution of non-*albicans* *Candida species* and their susceptibility to various antifungals among candidemia patients.

**Materials and Methods:**

A total of 123 confirmed Candida blood culture episodes from January 2011 to June 2022 were analyzed by retrospective laboratory-based observation. *Candida species* identity and the *in vitro* activity against antifungal drugs determined by guidelines from the Clinical and Laboratory Standard Institute (CLSI)

**Results:**

Most candidemia were caused by non-*albicans Candida species*, including *Candida parapsilosis* (37.4%), Candida tropicalis (17.1%), *Candida glabrata* (13.0%), *Candida guilliermondii* (3.2%) and others (4.8%). Meanwhile *Candida albicans* was found in 24.4% of cases. Among the patients, 57.7% were males and 68.3% were admitted to critical care with an age range of ≤ 28 days and 90 years. The pattern of *in vitro* susceptibility showed that 91.9% of the *Candida strains* were susceptible to amphotericin B, 89.3% to flucytosine, 97.3% to fluconazole, 98.3% to voriconazole, and 97.9% to echinocandins.

**Conclusion:**

Antifungal drug resistance was rare in our observation. The wide range of antifungal activities encourages management to carry out epidemiological surveillance in order to follow the dynamics of candidemia and influence the choice of therapeutic management for at-risk patients.

## Introduction

Blood stream candidiasis has become a major cause of morbidity and mortality in the healthcare setting, and changes in the epidemiology of Candida infections are currently occurring (Tragiannidis *et al.*, 2012). Candidemia, which is a form of invasive candidiasis, plays a significant role in healthcare-associated infections (HAIs), leading to increased length of stay, higher hospitalization costs, and elevated morbidity and mortality among patients (Horn *et al.*, 2009).

The epidemiology of candidemia varies according to geographies, patient characteristics, and the hospital system. According to Rodrigues (2021) and Mursinah (2016), risk factors associated with candidemia include the use of immunosuppressant agents such as corticosteroids, broad-spectrum antibiotics, and increase in invasive procedures in medical practice, especially in patients with hematological diseases, neutropenia, diabetes mellitus.

The incidence of invasive candidiasis varies in different countries ranging from 3.4% to 5.79%. The crude mortality rate of candidemia during the hospital admission was 72.2%. Some data in North America and Europe show the mortality rate of candidemia cases is more than 50% (Canela *et al.*, 2018). A study conducted in Indonesia reported that 50% of sepsis patients had candidemia during the hospitalization (Mursinah, *et al.*, 2016).

In recent years, there has been a shift in the causative agent of invasive candidiasis infection from *Candida albicans* to non-*albicans* *Candida* species such as *Candida parapsilosis*, *Candida tropicalis* and *Candida glabrata* (Doi *et al.*, 2016). Thus, good knowledge and understanding regarding the local epidemiology of invasive candidiasis is important to offer adequate case for this infection such as candidemia. In this observational study, we retrospectively reviewed candidemia cases from 1^st^ January 2011 to 30^th^ June 2022 at the Siloam Teaching Hospital, conducted an antifungal susceptibility testing for available clinical isolates, and compared the results with other previous studies to clarify changes in the epidemiology of candidemia.

## Material and Methods

In this study, Candida isolates were obtained retrospectively from the laboratory database of Siloam Teaching Hospital in Banten Province, Indonesia. A candidemia episode was defined based on the results of Candida isolation on blood culture. Chromogenic agar (BD CHROMagar® Candida, France) and Sabouraud dextrose agar were used to isolate the organisms. Identification and susceptibility testing against various antifungals were done through the Vitex2® Compact System (bioMérieux, France), whereas *in vitro* activity was determined according to the guidelines of the CLSI.

Antifungals like amphotericin B, fluconazole, voriconazole, 5-fluorocytosine, and micafungin were tested. Control strains were *C. parapsilosis* ATCC 22019 and *C. krusei* ATCC 6258. The minimum inhibitory concentration (MIC) was interpreted according to species-specific clinical breakpoints as established by CLSI for all types of antifungal agents (Pfaller and Diekema, 2012).

This study received approval from the local institutional review board. Given the retrospective and observational design of the study, written consent is not required.

## Results

This study analyzed 123 confirmed episodes of Candida blood cultures. According to Table 1, 57.7% of the cases were male, and 68.3% were admitted to critical care, spanning an age range from ≤ 28 days to 90 years. Table 1 demonstrates that over an 11.5-year observation period, species other than *Candida albicans* (76.6%) were the main causes of candidemia, including *Candida parapsilosis* (37.4%), *Candida tropicalis* (17.1%), *Candida glabrata* (13.0%). and others (8.1%).

**Table 1:** General Characteristics of Candidemia from Clinical Isolates, 2011-June 2022

	Frequency (n)												Total (n%)

	2011	2012	2013	2014	2015	2016	2017	2018	2019	2020	2021	January-June 2022	
Gender													
Male	2	3	10	10	6	7	5	4	7	7	7	3	71 (57.7)
Female	0	1	3	6	5	7	4	1	9	5	10	1	52 (42.3)
Age (mean, year)	84	43	47	49	49	43	45	40	28	41	64	49	48.5
Ward													
Inpatient	1	1	3	3	2	5	4	3	4	5	8	-	39 (31.7)
Intensive Care	1	3	10	13	9	9	4	2	12	7	9	4	84 (68.3)
Type of Candida													
*Candida albicans*	1	2	3	8	1	3	2	0	3	4	3	0	30 (24.4)
*Candida parapsilosis*	0	1	3	6	7	7	1	2	9	3	5	2	46 (37.4)
*Candida tropicalis*	1	0	2	2	1	2	6	0	1	4	2	0	22 (17.1)
*Candida glabrata*	0	0	2	0	2	2	0	1	2	1	4	2	16 (13.0)
*Candida guiliermondii*	0	0	1	0	0	0	0	2	0	0	0	1	4 (3.2)
*Candida krusei*	0	1	0	0	0	0	0	0	0	0	1	0	2 (1.6)
*Candida famata*	0	0	2	0	0	0	0	0	0	0	0	0	2 (1.6)
*Candida ciferii*	0	0	0	0	0	0	0	0	1	0	0	0	1 (0.81)
*Candida sake*	0	0	0	0	0	0	0	0	0	0	1	0	1 (0.81)

**Figure 1:**
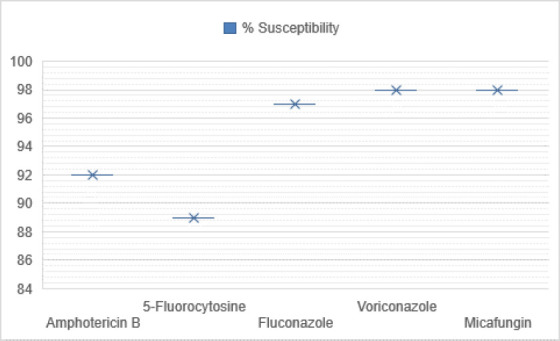
The susceptibility of various antifungal, 2011-June 2022

**Figure 2:**
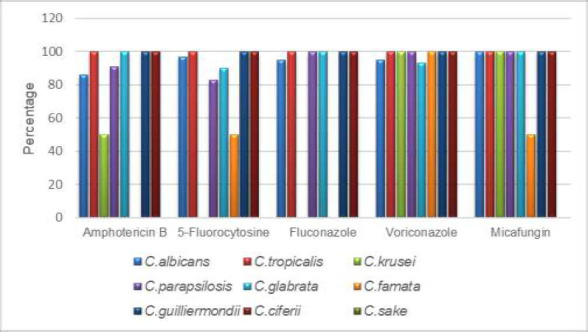
Percentage of antifungal susceptibility pattern towards various *Candida species*, 2011-June 2022

*In vitro* activity showed that 91.9% of Candida strains were susceptible to amphotericin B (MIC ≤ 0.25-1 mg/ml), and 89.3% to 5-flucytosine (MIC ≤ 1-4 mg/ml). Overall susceptibility to fluconazole, voriconazole and micafungin was ≥ 97.3%, 98.3%, and 97.9% with MICs ≤ 1-8 mg/ml, 0.06-2, and 0.12-1 mg/ml, respectively (Figure 1). Echinocandin, in this case micafungin has the best susceptibility level against various *Candida albicans* and non-*albicans Candida* species, except for *Candida famata* (50%). The azole group, both fluconazole and voriconazole also had a good susceptibility level, 95-100% (Figure 2).

## Discussion

The Candida non-*albicans* group has recently emerged as an important opportunist pathogen group, which possesses extracellular hydrolytic enzymes, biofilm formation, coagulase production, and phospholipase enzyme activity as virulence factors that contribute to pathogenicity and infection (Deorukhkar *et al.*, 2014). The expression of these virulence factors varies depending on species, geographic origin, type and site of infection, and host reaction. Thus, it also influences the pathogenicity and profile of the infecting candida.

In this present study, we uncovered 123 candidemia episodes, 22/123 (17.9%) of which were children with 12/22 (54.5%) of them classified as neonates. These results are in agreement with a previous study in Spain which found that 32% of the population were children and 25% of them were neonates (Viudes *et al.*, 2002). Candidemia is a severe illness among immunocompromised people with comorbidities such as malignancy, hematological disorder, transplant and steroid administration; and use of medical devices. or prolonged use of broad-spectrum antibiotics (Antoniadou *et al.*, 2003).

A multicenter study from America and Spain found that the global mortality rate due to Candida bloodstream infection is high in adults, ranging from 38.9% to 71%, compared to 29% in children. Three-month survival was better in the pediatric population than in adults (76% vs 54%; p<0.001) (Pappas *et al.*, 2003). Although our study did not observe the Candidemia mortality rate in children, another study found no significant difference in crude mortality for *Candida albicans* and non-*albicans Candida* (Cleveland *et al.*, 2012) Candidemia is a significant burden among critically ill patients. In India, it was observed that 65.2% of adults were infected with known risk factors, as evidenced after 8 days of admission to the intensive care unit, with a lower APACHE II score at admission (median 17.0; mean ± SD 17.2 ± 5.9; interquartile range 14–20). The highlights of those study were the various spectrum of Candida (31 *Candida species*) that cause candidemia and the high isolation rate from *Candida tropicalis* (41.6%) (Chakrabarti *et al.*, 2015).

In our study, non-*albicans Candida* species, as well as *Candida parapsilosis* (34.5%), *Candida tropicalis* (22.2%) represented the predominant isolated species. This result differs from a previous study in Italy, as noted in the report that *Candida albicans* was significantly the etiologic pathogen. The highest prevalence of candidemia in our study was comparable to previous observations in Atlanta, USA with a 61% outcome in the intensive care unit (ICU). Similarly, the rate is highest among patients aged 65 years (Barchiesi *et al.*, 2015). This report reveals a major shift to adulthood in the epidemiology of candidemia between certain age groups.

Over the past years, *Candida albicans* have become the predominant pathogen of candidemia worldwide. While *C. albicans* is still considered as the main etiologic pathogen causing candidemia, increasing rates of candidemia due to *C. tropicalis, C. parapsilosis, C. glabrata*, and *C. krusei* have been reported around the world. In this regard, *Candida glabrata* is frequently reported in Northern Europe and many US medical centers (Pinhati *et al.*, 2016). On the other hand, *Candida parapsilosis* and *Candida tropicalis* becoming the most common non-Candida albicans species associated with fungemia in Latin America and Asia (Morii *et al.*, 2014). Of note, a recent multicenter study in the US found that *C. parapsilosis* was the second most common non-*Candida albicans* species encountered (Pinhati *et al.*, 2016).

The susceptibility to various antifungals in our study were in agreement with reports from other regions, such as Latin America and Kuwait (Nucci *et al.*, 2013). The overall susceptibility to *Candida albicans* and non-*Candida albicans* species is still high, ranging from 89.1% to 97.8%. In a study conducted in Brazil, it was reported that decreased susceptibility to fluconazole occurred in 5% of isolates, and 1% of them were resistant. In addition, there was a linear correlation between the MIC of fluconazole and voriconazole (r = 0.54 and P < 0.001 [Spearman’s rho]) (Colombo *et al.*, 2006).

Our study provided much-needed information regarding the profiles of fungal pathogens from bloodstream infections and their antifungal susceptibility within the broader context of global literature. Although infrequent, resistant fungal pathogens threaten the tremendous patient health benefits that have been achieved by various antifungals. A potential limitation of our study that should be acknowledged is due to its retrospective nature, it may be influenced by selection bias. Another limitation is that we used laboratory data from a single center for the study, lacking the inclusion of pertinent clinical data. Therefore, longitudinal, multi-center studies with larger sample sizes are needed to validate the findings of our study.

## Conclusion

Resistance of antifungal drugs was infrequent in our study. The diversity of antifungal activities encourages the management to conduct epidemiological surveillance, in order to follow the dynamics of candidemia and influence the selection of therapeutic management for patients at risk.

### Conflict of Interests:

The authors declare that there is no conflict of interest associated with this study.

**List of Abbreviations used in the text:**
CLSI, Clinical and Laboratory Standard Institute,
HAIs, Health Care-Associated Infections
MIC, Minimum Inhibitory Concentration,
C, Candida,
APACHE II, Acute Physiology and Chronic Health Evaluation II

## References

[1] Antoniadou A, Torres H. A, Lewis R. E, Thornby J, Bodey G. P, Tarrand J. P, Kontoyiannis D. P (2003). Candidemia in a tertiary care center:in vitro susceptibility and its association with outcome of initial antifungal therapy. Medicine.

[2] Barchiesi F, Orsetti E, Gesuita R, Skrami E, Manso E, Candidemia Study Group (2015). The Candidemia study group:epidemiology, clinical characteristics, and outcome of candidemia in a tertiary referral center in Italy from 2010 to 2014. Infection.

[3] Canela H. M. S, Cardoso B, Vitali L. H, Coelho H. C, Martinez R, Ferreira M. E. S (2018). Prevalence, virulence factors and antifungal susceptibility of *Candida spp*. isolated from bloodstream infections in a tertiary care hospital in Brazil. Mycoses.

[4] Chakrabarti A, Sood P, Rudramurthy S. M, Chen S, Kaur H, Capoor M, Mendiratta D (2015). Incidence, characteristics and outcome of ICU-acquired candidemia in India. Intensive Care Medicine.

[5] Cleveland A. A, Farley M. M, Harrison L. H, Stein B, Hollick R, Lockhart S. R, Chiller T. M (2012). Change in incidence and antifungal drug resistance in candidemia:results from population-based laboratory surveillance in Atlanta and Baltimore, 2008-2011. Clinical Infectious Diseases.

[6] Colombo A. L, Nucci M, Park B. J, Nouér S. A, Arthington-Skaggs B, da Matta D. A, Morgan J, Brazilian Network Candidemia Study (2006). Epidemiology of candidemia in Brazil:a nationwide sentinel surveillance of candidemia in eleven medical centers. Journal of Clinical Microbiology.

[7] Deorukhkar S. C, Saini S, Mathew S (2014). Virulence factors contributing to pathogenicity of *Candida tropicalis* and its antifungal susceptibility profile. International Journal of Microbiology.

[8] Doi A. M, Pignatari A. C. C, Edmond M. B, Marra A. R, Camargo L. F. A, Siqueira R. A, Colombo A. L (2016). Epidemiology and microbiologic characterization of nosocomial candidemia from a Brazilian National Surveillance Program. PLoS ONE.

[9] Horn D. L, Neofytos D, Anaissie E. J, Fishman J. A, Steinbach W. J, Olyaei A. J, Webster K. M (2009). Epidemiology and outcomes of candidemia in 2019 patients:data from the prospective antifungal therapy alliance registry. Clinical Infectious Diseases.

[10] Morii D, Seki M, Binongo J. N, Ban R, Kobayashi A, Sata M, Tomono K (2014). Distribution of *Candida species* isolated from blood cultures in hospitals in Osaka. Japan J Infect Chemother.

[11] Mursinah M, Ibrahim F, Wahid M. H (2016). Risk factors and scoring systems for patients with candidemia at a tertiary hospital in Jakarta, Indonesia. Acta Med Indones-Indones J Intern Med.

[12] Nucci M, Queiroz-Telles F, Alvarado-Matute T, Tiraboschi I. N. D. S, Cortes J, Zurita J, Guzman-Blanco M (2013). Epidemiology of candidemia in Latin America:a laboratory-cased survey. PLoS ONE.

[13] Pappas P. G, Rex J. H, Lee J, Hamill R. J, Larsen R. A, Powderly W, Dismukes W. E, NIAID Mycoses Study Group (2003). A prospective observational study of candidemia:epidemiology, therapy, and influences on mortality in hospitalized adult and pediatric patients. Clinical Infectious Diseases.

[14] Pfaller M. A, Diekema D. J (2012). Progress in antifungal susceptibility testing of *Candida spp*. by use of Clinical and Laboratory Standards Institute broth microdilution methods, 2010 to 2012. J. Clin. Microbiol.

[15] Pinhati H. M. S, Casulari L. A, Souza A. C. R, Siqueira R. A, Damasceno C. M. G, Colombo A. L (2016). Outbreak of candidemia caused by fluconazole resistant *Candida parapsilosis* strains in an intensive care unit. BMC Infectious Diseases.

[16] Rodrigues D. K. B, Bonfietti L. X, Garcia R. A, Araujo M. R, Rodrigues J. S, Gimenes V. M. F, Melhem1 M. S. C (2021). Antifungal susceptibility profile of candida clinical isolates from 22 hospitals of São Paulo State, Brazil. Brazilian Journal of Medical and Biological Research.

[17] Tragiannidis A, Fegeler W, Rellensmann G, Debus V, Müller V, Hoernig-Franz I, Groll A. H (2012). Candidemia in a European Pediatric University Hospital:A 10-year Observational Study. Clinical Microbiology and Infection.

[18] Viudes A, Pemán J, Cantón E, Ubeda P, López-Ribot J. L, Gobernado M (2002). Candidemia at a tertiary-care hospital:epidemiology, treatment, clinical outcome and risk factors for death. European Journal of Clinical Microbiology and Infectious Diseases.

